# Potential role of resveratrol in prevention and therapy of diabetic complications: a critical review

**DOI:** 10.29219/fnr.v68.9731

**Published:** 2024-04-30

**Authors:** Mehdi Koushki, Masoumeh Farahani, Reyhaneh Farrokhi Yekta, Naghmeh Frazizadeh, Parisa Bahari, Negin Parsamanesh, Hossein Chiti, Somayeh Chahkandi, Mohammadjavad Fridoni, Nasrin Amiri-Dashatan

**Affiliations:** 1 Department of Clinical Biochemistry, School of Medicine, Zanjan University of Medical Sciences, Zanjan, Iran; 2 Skin Research Center, Shahid Beheshti University of Medical Sciences, Tehran, Iran; 3 Proteomics Research Center, Shahid Beheshti University of Medical Sciences, Tehran, Iran; 4 Zanjan Metabolic Diseases Research Center, Zanjan University of Medical Sciences, Zanjan, Iran; 5 Department of Anatomy, School of Medicine, Zanjan University of Medical Sciences, Zanjan, Iran

**Keywords:** resveratrol, diabetes mellitus, nephropathy, retinopathy, neuropathy, diabetic foot ulcer, cardiomyopathy

## Abstract

**Background:**

Diabetes mellitus (DM) is a category of metabolic conditions affecting about 5% of people worldwide. High mortality associated with DM is mostly due to its severe clinical complications, including diabetic nephropathy, retinopathy, neuropathy, and cardiomyopathy. Resveratrol (RSV) is a natural, biologically active polyphenol known to have various health-promoting effects in animal models and humans.

**Objective:**

In this review, we have reviewed the preventive and therapeutic role of RSV on diabetes complications with emphasis on its molecular mechanisms of action.

**Methods:**

To prepare this review, all the basic and clinical available literatures regarding this topic were gathered through electronic databases, including PubMed, Web of Science, Scopus, and Google Scholar. Therefore, we summarized previous studies that have evaluated the effects of RSV on diabetic complications and their mechanisms. Only English language studies published up to January 2023 were included in this review.

**Results:**

RSV improves glucose homeostasis, decreases insulin resistance, induces autophagy, regulates lipid metabolism, protects pancreatic β-cells, ameliorates metabolic disorders, and increases the GLUT4 expression. These effects induced by RSV are strongly associated with ability of this polyphenol agent to elevation expression/activity of AMP-activated protein kinase and Sirtuin 1 in various organs of diabetic subjects, which leads to prevention and therapy of diabetic complications. In addition, antioxidant and anti-inflammatory properties of RSV were reported to be involved in its action in diabetic complications, such as retinopathy and nephropathy.

**Conclusion:**

RSV is a promising compound for improving diabetic complications. However, the exact antidiabetic mechanisms of RSV need to be further investigated.

## Popular scientific summary

- Nephropathy, retinopathy, and foot ulcer are considered as diabetic complications.
- Resveratrol may act against diabetic complications via modulating signaling pathways.
- This review provides evidence that resveratrol may benefit the management of T2DM.

Diabetes mellitus (DM) is a main public health problem and devastating condition with an increasing prevalence over the past few decades. DM is a group of chronic metabolic abnormalities that are defined by hyperglycemia resulting from the lack of insulin production, resistance to insulin action, or even both (1). According to the International Diabetes Federation, the diabetic patients are expected to exceed 640 million by 2040 (2). In addition, it is estimated that about half of the diabetic patients are not aware of their disease and therefore are more exposed to its complications (3). The traditional complications of DM include cardiomyopathy, peripheral neuropathy, retinopathy, and nephropathy. Moreover, along with advancements in the management of DM, a connection has been revealed between DM and risk of emerging complications such as cancer (4), infectious diseases (5), cognitive disorders (6), liver disease (7), and sleep disorders (8). DM patients are also at increased risk for COVID-19 infection (9). Complications of DM are common in both types 1 and 2 diabetes, which are the main causes of morbidity and mortality among these patients. The chronic complications of DM in general are divided into microvascular and macrovascular, the first one with higher prevalence than the second (10). These progressive and fatal complications make diabetes a more serious problem than other diseases.

Continuous use of synthetic drugs usually leads to side effects, and thus, the request has gone toward non-/less-toxic herbal-derived drugs for several diseases, such as diabetes (11), cancer (12), Non-alcoholic fatty liver disease (NAFLD) (13), infection diseases (11, 14), and inflammation-related diseases (15–17). The World Health Organization (WHO) has rostered a total of 21,000 plants that are used worldwide, among which, more than 400 plants are available for diabetes therapy (18). Nevertheless, only a few numbers have been evaluated to assess their efficacy. Recent evidences strongly suggest that the resveratrol (RSV) has protective effects against diabetes and its complications. The protective mechanisms of RSV involve several signaling pathways regulations, including the inhibition of oxidative stress (OS) and hyperinflammation, improvement of insulin action, induction of autophagy, regulatory effect on lipid metabolism, increasing GLUT4 expression, and activation of Sirtuin 1 (SIRT1)/Adenosine Monophosphate Activated Protein Kinase (AMPK) signaling axis. In this review, potential preventive and therapeutic roles of RSV on diabetes complications have been widely discussed.

## Physical and physiological properties of resveratrol

**Fig. 1:**
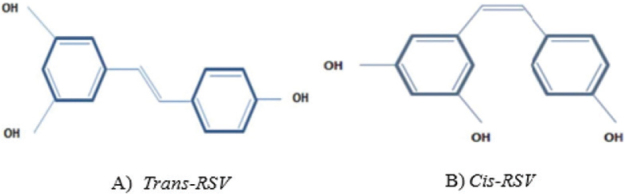
Chemical structure of resveratrol. (A) Trans-RSV and (B) Cis-RSV (11).

RSV (3,4′,5-trihydroxy-*trans*-stilbene) is a polyphenolic phytoalexin with 228.25 g/mol molecular weight that is found in grapes, peanuts, berries, groundnut, spruce, and mulberries. It has numerous beneficial antioxidants, anti-inflammatory, anticarcinogenic, antidiabetic, neuroprotective, cardioprotective, and antiaging characteristics in humans (19–21). The basic structure of RSV consists of two phenolic rings bonded together by a double styrene bond. This bond is responsible for the isometric cis- and trans-forms of RSV (Fig. 1). The trans-isomer is prevailing, and stable form of RSV and cis-isomer can appear when the trans-isoform is exposed to solar or ultraviolet radiation (at a 254 or 366 nm wavelength) (22). The low solubility of RSV is due to its chemical structure, which also affects its absorption. Different approaches are used to increase the absorption of RSV. For instance, acetylation of RSV can improve its absorption and its cellular permeability without reducing or losing its activity (23). The passive diffusion and complexes formation with membrane transporters like integrins are the underlying mechanism of intestinal absorption of RSV. Due to RSV is poorly soluble in water, it must be bonded to plasma proteins to distributed in whole body and be bioavailable. In the bloodstream, RSV can be recognized in three forms, including glucuronide, sulfate, or free. Of these, the free form has the ability to bind to albumin, hemoglobin (Hb), and low-density lipoprotein (LDL), and then enters into the cells through albumin and LDL receptors (24). According to investigations, the RSV–albumin complex is more stable than RSV–Hb, suggesting that serum albumin has a high affinity for RSV playing a critical role in distribution and bioavailability of RSV (25). Studies have revealed that bioavailability of RSV possesses high orally absorption but quick and extensive metabolism resulting in only low levels of unchanged RSV in the systemic circulation (26). Metabolism of RSV in humans revealed that about 70% of orally administered RSV (25 mg) is quickly (<30 min) absorbed and metabolized in less than 30 min and a half-life of 9–10 h (27). RSV has high metabolism and undergoes phase I and phase II metabolism in the liver and intestinal epithelial cells rapidly after administration resulting in glucuronic acid and sulfate conjugation metabolites that keep some biological function (28). Lipophilic properties of RSV lead to a high absorption. Nevertheless, absorption of this compound can differ depending on its usage way and the kind of food that contains RSV (29). Many studies report high beneficial effects of RSV; however, its low bioavailability might decrease the efficacy of RSV. Therefore, *the findings of in vitro studies* must be interpreted with caution. On the other hand, the biological effects of RSV can be justified with enterohepatic recirculation of its metabolites, followed by its deconjugation and reabsorption in the small intestine (30). In addition to beneficial effects of RSV and its metabolites, they may also generate cytotoxic or immune-toxic effects (31). A study (32) reported that RSV is not irritating to the skin and eyes and also has no genetic toxicity. They repeated subchronic toxicity tests again after 90 days and observed that RSV did not have any toxicity at the maximum dose of 700 mg/(kg·d). These findings indicated that RSV is non-toxic and safe. In other study, Hebbar et al. found that at 1.0 and 3.0 g/(kg·d), female and male rats showed dehydration, dyspnea, kidney toxicity, and elevated serum liver enzymes (33).

## Literature search strategy

The aim of this article was to review all the literature on RSV and its beneficial effects in diabetes-related complications in the last two decades. For this purpose, we conducted a literature search in PubMed, Web of Science, Scopus, and Google Scholar up to January 2023. All basic (in vitro and animal models) and clinical studies selected for further evaluation in detail using search terms in title and abstract. The following search terms were used to identify relevant studies: (‘Resveratrol’ OR ‘3, 5, 4′-trihydroxystilbene’) AND (‘Diabetes’ OR ‘Diabetes Mellitus’ OR ‘T1DM’ OR ‘T2DM’) AND (‘Diabetic Complications’ OR ‘Nephropathy’ OR ‘Retinopathy’ OR ‘Neuropathy’ OR ‘Cardiomyopathy’ OR ‘Myopathy’ OR ‘Diabetic Foot Ulcer’). Given the large volume of articles on RSV, therefore, we focused on the most impactful articles published in recent years. Moreover, clinicaltrials.gov website was searched to select all clinical trials concerning the effect of RSV on diabetic complications. In addition, only articles written in English and original investigations were included in this review.

## Beneficial effects and molecular mechanisms of resveratrol in diabetes

**Fig. 2:**
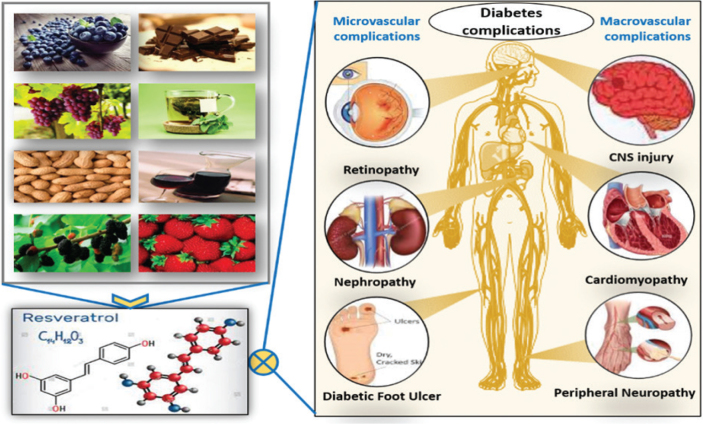
Dietary sources, chemical structure, and beneficial effects of resveratrol on diabetes complications.

Types 1 and 2 are the main classes of DM. Type 1 diabetes is insulin-dependent, which results from autoimmune demolition of pancreatic beta cells. Type 2 diabetes (T2DM) is the most common form, and about 90% of all diabetic patients are affected by this type. In patients with T2DM, insulin resistance (IR) or insulin secretion deficiency occurs during disease. It has recently been suggested that inflammation and OS are involved in worsening of IR and β-cell failure in T2DM (34). Normoglycemia and preservation of pancreatic β-cells are the main treatment issues in diabetic patients (35). Recent data indicate that RSV, as a natural compound, exerts various beneficial effects on diabetes, which are extensively studied in vitro and in animal models. RSV (3, 5, 4′-trihydroxystilbene) is a phenolic micronutrient agent, which is produced naturally by about 70 various types of plant species (including grapes, berries, chocolate, peanuts, and pines). RSV was initially defined as an antibiotic formed against environmental stresses, such as infection, radiation, and pathogenic conditions (36). Studies conducted in animal models reported that the only side effect of RSV consumption was the onset of diarrhea after receiving 200 mg of RSV daily, which was not associated with clinical complications. Totally, this compound appears to have a high safety in vivo (37). Most studies of the RSV effect on diabetes were animal studies, which demonstrated multiple protective effects for RSV in dealing with diabetes. The protective effects of RSV on diabetes are summarized in Fig. 2. Most important beneficial effects of RSV include enhancement of glucose uptake and its metabolism, improvement of pancreatic beta-cell protection, and improvement of IR in muscle, liver, and adipose tissue (38–40).

Animal studies suggested that RSV could preserve glucose in the normal range and protect pancreatic β-cells in type 1 diabetics. In addition, RSV enhances antioxidant capacity of pancreatic beta cells through elevating antioxidant enzymes (including superoxide dismutase, catalase, and glutathione S-transferase) (41). RSV also inhibits Poly (ADP-Ribose) polymrase (PARP) enzyme and moderates hyperglycemia in streptozotocin (STZ)-induced degeneration of the β-cells (42). Another protective mechanism of RSV in type 1 diabetes appears in skeletal muscle. Chang et al. reported that RSV alleviates skeletal muscle dysfunction in animal models with type 1 diabetes (43). In addition, RSV stimulates mitochondrial biogenesis in skeletal muscle that leads to increased lipid metabolism in diabetic models. RSV also has anti-inflammatory effects and reduces levels of proinflammatory cytokines, such as nuclear factor kappa-light-chain-enhancer of activated B cells (NF-κB), interleukin (IL)-1β, and IL-6 and also reduces OS (43, 44).

GLUT4, a main regulatory factor for glucose transport, plays an important role in the glucose uptake of skeletal muscle cells. The decreased expression of GLUT4 protein has been reported in diabetic models. RSV consumption in the amount of 3 mg/kg for 7 days can increase liver expression of GLUT4 and GLUT2 in the diabetic rats (45); however, the precise mechanism of GLUT4 action remains unclear. A major part of investigations considered that the increment of GLUT4 and GLUT2 is associated with the activation and phosphorylation of phosphatidylinositol 3-kinase (PI3K) and AMPK by RSV, respectively (46, 47). Additionally, RSV induces alterations in metabolism enzyme levels, which can increase glycolysis and glycogen synthesis in the skeletal muscles (48). RSV is also found to decrease gluconeogenesis and increase liver glycogenesis that can reduce the hepatic glucose output. Moreover, it has a protective effect against liver abnormalities (34). The antidiabetic effects of RSV are well described in animal models of diabetes (34, 42, 49). Some of the possible mechanisms of RSV action in animal diabetic models are presented in Fig. 2. Preservation of islet β-cells and release of insulin from β-cells are another beneficial effect of RSV on diabetes. In this regard, SIRT-1 and AMPK were presented as the most important molecular targets, by which RSV mediates its protective effects against pancreatic β-cell dysfunction and improves insulin sensitivity (50, 51). The activation of these targets by RSV leads to further processes, such as decreased lipid accumulation, inflammation, and oxidative damage, and finally improvement of insulin action in diabetic patients. In addition, increased phosphorylation of insulin receptor, Insulin receptor substrate-1 (IRS-1) expression, and phosphorylation of Akt have been proposed as potential mechanisms in RSV action (52). Cheng et al. reported that RSV promotes phosphorylation of nuclear factor erythroid 2-related factor-2 (Nrf2) in mouse models, which makes this hypothesis stronger that the RSV might be helpful in the diabetes therapy by the protective effects against dysfunction of beta-cells. The regulation of inflammatory factors has an important role in the protective effects of RSV in the islet cells. Tian et al. conducted an animal study and reported that RSV enhances AMPK phosphorylation and SIRT1 protein levels and inhibits the extreme activation of NF-κB pathway, thereby decreases the inflammatory cytokines levels (53).

**Fig. 3:**
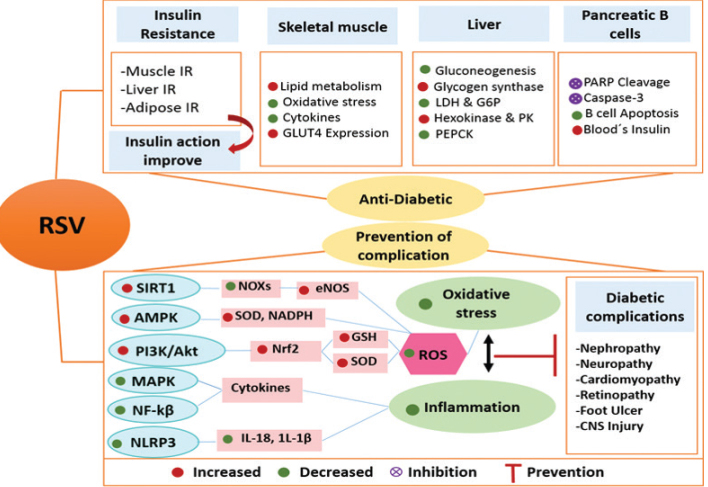
Mechanism of potential action of resveratrol against diabetes and its related complications.

IR is defined clinically as the inability of a known quantity of exogenous or endogenous insulin to increase glucose uptake and utilization. According to studies, the administration of RSV at a dose of 2.5−400 mg/kg for 1–6 months improves the insulin sensitivity in T2D individuals and animal models. In a meta-analysis, the effect of RSV on glucose control and insulin sensitivity for 11 randomized controlled trials was investigated. The authors concluded that RSV significantly improves glucose control and insulin sensitivity in diabetic patients but does not affect glycemic measures in non-diabetic individuals (54). Generally, diabetes management encompasses main approaches, including blood glucose reduction, protection of beta cells from dysfunction, and insulin action improvement. The literature findings showed that the beneficial effects of RSV in relation to diabetes include all of these aspects (Fig. 2). Potential molecular mechanisms of RSV action against diabetes and its related complications are illustrated in Fig. 3.

## Resveratrol effects on diabetic complications

### RSV role in diabetic nephropathy

Diabetic nephropathy (DN) is a major and serious long-term diabetes complication that is indicated by glomerular hypertrophy, renal fibrosis, proteinuria, reduced glomerular filtration, and weakened kidney function and is the ultimate cause of end-stage renal disease (ESRD). Pathologically, DN is defined by basal and glomerular membrane thickening, extracellular matrix (ECM) components accumulation, glomerulosclerosis and related progressive mesangial development, and reduction in glomerular filtration surface. Several mechanisms are involved in DN progression, such as kidney hemodynamic alterations, lipid disorders, OS, mitogen and polyol protein kinase activation, and rise in the non-enzymatic glycosylation of proteins signaling pathways. These conditions in the kidneys finally lead to ESRD, and dialysis or transplantation is needed for therapy (55, 56). Wen et al. showed the improved outcome of RSV on kidney dysfunction in STZ-induced diabetic rats. The rats received 20 mg/kg RSV per day for 8 weeks intraperitoneally. The results demonstrated that RSV remarkably reduced urinary albumin excretion, kidney mass and interstitial fibrosis, and glomerular basement membrane thickness. Various evidences have shown the elevated antioxidant defense and reduction of reactive oxygen species (ROS)-induced inflammation in the kidney by RSV, which are accompanied by altered regulations in diverse cellular signaling cascades, including AMPK pathway, NF-κB, Mitogen-activated protein kinase (MAPK), and sphingosine kinase pathway (57).

In the C57 diabetic animal model, RSV was administered in a dose of 40 mg/kg per day for 12 weeks by gastric perfusion. RSV efficiently attenuated DN and reduced blood glucose levels by preventing the expression of nicotinamide adenine dinucleotide phosphate oxidase 4 (NOX4) in kidney tubular epithelial cells by AMPK activation (58). This pathway declines NOX4-derived intracellular ROS. Generally, RSV decreases kidney fibrosis and restores kidney function in mice by modulating AMPK/NOX4/ROS signaling. Park et al. indicated that RSV elevated adiponectin receptor-1 (AdipoR1) and AdipoR2 expression and activated the AMPK-SIRT1-peroxisome proliferator-activated receptor (PPAR)-gamma coactivator (PGC)-1alpha axis, and peroxisome PPAR-alpha, thus reduced lipotoxicity and improved OS, cell apoptosis, and endothelial dysfunction in DN (59). Furthermore, RSV prevents diabetes-induced nephritis and glomerular membrane cell proliferation by preventing the Akt/NF-κB pathway. In patients with dominant type 2 DN, oral RSV administration exerts beneficial effects on glomerular function by controlling inflammation factors (60). DM microvascular complications are mostly recognized by hyperglycemia, which is known as the primary feature affected by DN. Evidences recommend that raised glucose flux could grow ROS production via the mitochondrial electron transport chain (61). The RSV capability to produce antioxidant protection against DN is shown in a number of cell culture reports and preclinical studies (62). For instance, in streptozotocin-induced DM, RSV therapy remarkably reduced pre-DN complications, such as creatinine, proteinuria, urea, and OS by antioxidant mechanisms. Kitada et al. demonstrated that RSV therapy improved kidney damage and reduced mitochondrial OS and biogenesis by manganese-superoxide dismutase regularization (MnSOD) in male db/db mice. Therefore, RSV can defend against kidney injury induced by the OS in DM by improving the antioxidant mechanism and declining lipid peroxidation (63). Oral therapy by RSV could meaningfully upgrade renal dysfunction and reduce the pro-inflammatory cytokines production, which was supplemented by an improvement in the antioxidant system by standardizing the Nrf2-Keap1 expression in (T2D) DM rats. RSV administration has been revealed to decline high-glucose-induced cell proliferation and fibronectin expression through c-Jun N-terminal kinase (JNK) and NF-κB blocking in mesangial cells (64). Furthermore, ROS was decreased by RSV in mesangial cells by blocking the NOX activity. In a T2D rat model, RSV (5 mg per day) improved kidney damage, which was indicated buy a considerable reduction in urinary protein and TGFβ1 expression and serum creatinine compared with the control group (65). It also caused a great development in the antioxidant system. Another study also showed normalization of kidney expressions of SIRT1, TGF-β1, fibronectin, forkhead box O (FOXO1), and NF-κB/p65 by RSV (65–67). Table 1 shows RSV nephron-protective roles in diabetes.

**Table 1:** Resveratrol nephron-protective studies in diabetes

Herb (dosage/duration)	Study type and model	Finding	Ref.
**In-vitro** **- 25μM** **- 24 h** **In-vivo** **-20 mg/kg/day** **- 8 weeks**	**In-vitro** Podocytes or endothelial cells **In-vivo** STZ-induced diabetic mice	**In-vitro Results** - Decreased high-glucose-induced VEGF and Flk-1 expression in cultured mouse glomerular podocytes and endothelial cells **In-vivo Results** - Decreased VEGF, Flk-1, and angiopoietin 2 - Increased Tie-2 in rat kidneys and kidney function	(57)
**In-vitro** **- 50 μM** **In-vivo** **- 20 mg/kg/day** **- 12 weeks**	**In-vitro** Human glomerular endothelial cells **In-vivo** C57BLKS/J db/m	**In-vitro Results** - Decreased high-glucose-induced apoptosis and oxidative stress in glomerular endothelial cells - Increased AdipoR1 and AdipoR **In-vivo Results** - Increased phosphorylation of AMPK and SIRT1 - Decreased downstream effectors FOXO1 and FOXO3a - Increased AdipoR1 and AdipoR2 in renal cortex, PPARγ coactivator-1α, and estrogen-related receptor-1α - Decreased sterol regulatory element-binding protein 1	(59)
**In-vivo** **- 10 mg/kg/day** **- 12 weeks** **In-vitro** **- 25 μM**	**In-vivo** STZ-induced diabetic rat **In-vitro** Rat mesangial cell	**In-vivo Results** - Decreased PAI-1, p-Akt/Akt ratio, and NF-κB in the kidneys, PCNA-positive cells in glomeruli **In-vitro Results** - Decreased proliferation of cell nuclear antigen-positive cells in glomeruli of diabetic mice	(60)
**- 10 μmol/L** **- 24 h**	**In-vitro** Renal mesangial cell	- Decreased high-glucose-induced mesangial cell proliferation and fibronectin - Decreased JNK and NF-κB activation, NAD(P)H oxidase activity elevation, and ROS production	(64)
**- 0.3% with** **Non-purified diet** **- 8 weeks**	**In-vivo** db/db mice	- Decreased urinary albumin excretion - Decreased mitochondrial oxidative stress and biogenesis in the kidneys	(63)
**- 5 mg/kg/day** **- 30 days**	**In-vivo** STZ-induced diabetic rats	- Normalized the OS, inflammatory markers, kidney expression of Nrf2/Keap1, and its downstream regulatory proteins in diabetes	(65)
**- 10 μmol/L**	**In-vitro** Renal mesangial cell	- Decreased high-glucose-induced ROS production and cell apoptosis - Increased SIRT1, PGC-1α, NRF1, mitochondrial transcription factor A, and respiratory chain complexes I and III activity	(64)
**In-vivo** **- 5 mg/kg/day** **In-vitro** **- 5 mg/kg/day** **- 24, 30, 36, and 42 h**	**In-vivo** STZ-induced diabetic rats **In-vitro** Rat renal proximal tubular cell line	**In-vivo Results** - Upregulation of SIRT1 expression - Downregulation of Atg7, Atg5, and LC3 proteins **In-vitro Results** - Increased antioxidant defense mechanism - Normalized kidney mRNA expressions of TGF-B1, FOXO1, fibronectin, NF-κB/p65, Nrf2, and Sirt1	(68)
**- 5–20 μmol/L** **- 48 h**	**In-vitro** Human proximal tubular cell line HK-2	- Decreased high-glucose-induced EMT, intracellular ROS, NOX1, NOX2, and ERK1/2	(69)
**- 1, 5, 10 mg/kg/day** **- 30 days**	**In-vivo** STZ-induced diabetic mice	- Decreased malondialdehyde, plasma glucose, and expression of RAGE - Increased antioxidant property and insulin concentrations	(70)
**RSV** **- 20 μmol/L** **In-vitro** **- 72 h** **In-vivo** **- 12 weeks**	**In-vitro** Mesangial cells line (HBYZ-1) **In-vivo** STZ-induced diabetic rats (single I.P. injection of 60 mg/kg STZ)	**In-vitro Results** - Increased FOXO1 and AdipoR1 in mesangial cells **In-vivo Results** - Increased AdipoR1, and kidney pathological marker - Decreased malondialedehyde, collagen IV, and fibronectin proteins	(71)
**In-vitro** **- 1, 5, and 50 ng/mL** **- 48 h** **In-vivo** **- 20 mg/kg/day** **- 12 weeks**	**In-vitro** NMS2 mesangial cells **In-vivo** C57BLKS/J db/db mice	**In-vitro Results** - Increased AMPK phosphorylation, SIRT1-PGC-1α/PPARα-ERR-1α-SREBP1 **In-vivo Results** - Decreased lipid - Increased AMPK phosphorylation, SIRT1-PGC-1α signaling and PPARα-ERR-1α–SREBP1	(72)
**- 30 mg/kg/day** **- 12 weeks**	**In-vivo** STZ-induced diabetic mice (single dose of 60 mg/kg STZ)	- Increased SIRT1, FOXO1 activity, and SOD activity - Decreased malondialdehyde, collagen IV, and fibronectin protein	(73)
**- 5 and 10 μmol/L** **- 48 h**	**In-vitro** Rat mesangial cell	- Decreased ROS production and mitochondrial superoxide generation in mitochondrial complex III activity in mesangial cells	(74)
**In-vivo** **- 10 mg/kg/day** **- 14 weeks** **In-vitro** **- 5, 10, and 20 μmol/L** **- 24 h**	**In-vivo** STZ-induced diabetic mice **In-vitro** Mesangial cells	**In-vivo Results** - Increased AMPK and kidney function - Decreased eukaryotic translation initiation factor 4E-BP1, phospho-ribosomal protein S6 (S6), plasma creatinine, and urinary albumin excretion **In-vitro Results** - Decreased high-glucose-induced dephosphorylation of AMPK and phosphorylation of 4E-BP1 and S6 - Decreased DNA synthesis and proliferation	(75)
**- 5 mg/kg** **- 0, 24, and 48 h**	**In-vivo** - Rabbit model contrast-induced nephropathy	- Modulating oxidant-antioxidant balance - Decreased vasoconstriction/blood cytotoxicity	(76)
**- N/A**	**In-vivo** - C57BL/6J mice fed with high-fat diet for 12 weeks	- Regulates the JAML/Sirt1 lipid synthesis pathway - Decreased lipid deposition in the kidney - Decreased diabetic kidney damage	(77)
**- 200 mg/day** **- 24 weeks**	Clinical trial Human with DM-type 2	- Improvement of glycemic control by reducing insulin resistance - RSV decreased diabetic associated complications	(78)
**- 10 μmol/L** **- 10 to 14 days**	**In-vitro** High-glucose-induced oxidative stress and apoptosis in podocytes	- Decreased ROS production and apoptosis - Increased respiratory chain complex I and III activity - Increased mitochondrial membrane potential	(79)
**- 30 mg/kg/ day**	**In-vivo** Diabetic mice	- Decreased proteinuria and malondialdehyde content - Increased MnSOD activity in renal cortex of diabetic mice - Increased SIRT1 and PGC-1α	(80)
**- RSV (20 mg/kg/ day) + insulin** **- 8 weeks**	**In-vivo** STZ-induced DM-type-1 renal damage rat	- Increased kidney cortex antioxidant enzyme activities and Na+/K+-ATPase - Decreased lipid peroxidation	(81)
**- 50 mg/kg/ day** **- 6 weeks**	**In-vivo** STZ-induced diabetic rats (50 mg/kg)	- Decreased ER stress, p-PERK, GRP78, ATF4, and CHOP	(45)
**- 5 mg/kg/ day** **- 90-day**	**In-vivo** STZ-induced diabetic rats (55 mg/kg)	- Decreased kidney hypertrophy and structural alternation like tubular atrophy, diffuse glomerulonephritis, mesangial expansion or shrinkage, and fibrosis - Decreased AGE accumulation, DNA damage, and oxidative stress	(82)
**- 25 μmol/L** **- 16 weeks**	**In-vivo** STZ-induced diabetic rats	- Increased SIRT1 deacetylase activity - Decreased acetylated-FOXO3a, oxidative stress, and decreased silencing SIRT1	(83)
**- 10 μmol/L** **- 12 weeks**	**In-vivo** C57BL/Ks rats	- Increased autophagy - Decreased apoptosis in podocytes	(84)
**- 10 μmol/L**	**In-vitro** Mouse podocytes cell lines	- Increased LC3-II/LC3-I - Decreased cleaved caspase expression	(85)
**In-vivo** **- 40 mg/kg/day** **- 12 weeks** **In-vitro** **- 20 μmol/L** **- 48 h**	**In-vivo** C57BL/KS db/db mice **In-vitro** NRK49F cells	**In-vivo Results** - Decreased kidney fibrosis and NOX4 - Increased p-AMPK **In-vitro Results** - Decreased high-glucose-induced cell proliferation, p-AMPK, and NOX4 expression	(58)

### RSV and diabetic retinopathy

Diabetic retinopathy is one of the most common diabetes complications, affecting the eyes. About 80% of people with diabetes encounter symptoms of diabetic retinopathy after 15 years, and more than 10% might be at risk of vision loss. OS, inflammation, angiogenesis, and neuro-glial dysfunction are implicated in the pathogenesis of diabetes-related retinopathy. RSV treatment modulates several physiological processes, including OS, inflammation, cell proliferation, apoptosis, or angiogenesis. Current findings have demonstrated the therapeutic potential of RSV in the treatment of eye diseases as an antioxidant, anti-inflammatory, antiapoptotic, antitumor, antiangiogenic, and vasorelaxant molecule (86–89). Kubota et al. demonstrated that RSV treatment can promote the activity of AMPK, inhibit the SIRT1 deactivation, and repress the NF-kB phosphorylation (90). RSV has been reported to prevent retinal neural apoptosis by the induction of RNA-dependent-protein-kinase (PKR)-associated protein X(RAX) (RAX/P-PKR) expression in the diabetic retina (91). Al-Hussaini et al. reported that trans-RSV treatment in diabetic rats increased both antiapoptotic and proapoptotic proteins. They also showed that trans-RSV can target the intrinsic apoptotic pathway in retinal pigment epithelium cells (RPE). RPE plays supporting roles in neural retina functions such as absorption of light, transport and recycling of retinoids, and phagocytosis of daily-shed photoreceptor outer segments. Diabetes induces adverse effects in RPE. Trans-RSV treatment in diabetic rats showed a protective outcome against diabetes-induced RPE defects by inhibiting the intrinsic apoptosis pathway (92). The antiapoptotic mechanism of RSV has been evaluated on retinal Müller cells of diabetic rats. Müller cells, as one of the primary glial cell types, play a significant role in maintaining the normal health of the retina. The findings of this study implied that RSV prevents diabetes-induced apoptosis in Müller cells through the miR-29b/SP1 pathway (93). Chen et al. assessed the effect of RSV on retinal apoptosis by the caspase-3 activity. RSV significantly decreased retinal augmentation of active caspase-3 level in the STZ-induced diabetic rats (94). Soufi et al. observed a substantial increase in NF-kB activity and apoptosis rate in the retinas of diabetic control and RSV-treated diabetic groups as compared with normal groups. RSV treatment significantly decreased these enhancements (95). In another study, Soufi et al. demonstrated that RSV treatment can decrease the apoptosis rate in the polymorphonuclear cells, retina, and some other tissues of diabetic rats (96).

Some other findings suggest the regulatory role of RSV in diabetes-induced retinal inflammation. Some proteins and relevant signaling pathways are involved in the anti-inflammatory effects of RSV in the diabetic retina. RSV significantly reduced the downregulation of occluding induced by diabetes and upregulation of high-mobility group box-1 (HMGB1), induced by diabetes, as well as the receptor for advanced glycation end (RAGE) products and breakdown of blood-retinal barrier (BRB) in the diabetic retina. HMGB1 is known as a key player of inflammation in the retina. There is a functional link between SIRT1, with anti-inflammatory properties, and HMGB1 in the regulation of BRB breakdown induced by diabetes. Activation of SIRT1 by RSV decreases the upregulation of HMGB1. Therefore, the protective effect of RSV against diabetes-induced retinal endothelial barrier dysfunction is associated with cross-talk between SIRT1 and HMGB1 (97). The findings of Chen et al., via gene ontology enrichment analysis, indicated that RSV-induced Paraoxonase 1 (PON1) may regulate retinal inflammation and damage in diabetic retinopathy. Their results suggested that RSV treatment reduces the levels of inflammatory factors IL-1β, IL-6, TNFα, vascular endothelial growth factor (VEGF), Interferon gamma (IFNγ), and Monocyte chemoattractant protein-1 (MCP-1) (94). Soufi et al. evaluated the anti-inflammatory effects of RSV, by measuring the plasma levels of TNFα and IL-6. The TNFα and IL-6 concentrations were higher in both diabetic control group and diabetic group treated with RSV compared to normal controls, and the application of RSV was shown to reduce these alterations (96). The present findings suggest the AMPK activator, RSV, prevents cellular and molecular mechanisms in diabetes-induced retinal inflammation, which is known to mediate NF-κB activation. Moreover, SRV stimulates SIRT1 activity, which was decreased in the diabetic retina. Application of RSV reverses SIRT1 deactivation along with NF-κB activation, indicating a potential relationship between SIRT1 deactivation and NF-κB activation in the regulation of diabetes-induced retinal inflammation (90). Soufi et al. studied the long-term anti-inflammatory characteristics of RSV in the retinas of diabetic rats. RSV treatment significantly inhibited the increase in proinflammatory cytokines, such as TNF-α, IL-6, and cyclooxygenase-2 (COX-2) (98). Losso et al. showed anti-inflammatory mechanism of RSV treatment on A spontaneously arising retinal pigment epithelia (RPE) cell line (ARPE-19) cells. They also reported the inhibitory activity of RSV on the accumulation of several pro-inflammatory markers, including VEGF, TGF-β1, COX-2, IL-6, and IL-8 in a dose-dependent manner. Protein Kinase C (PKC)-β activation was also reduced, and the expression levels of Cx43 protein and GJIC were increased in the presence of RSV (99).

Hyperglycemia leads to the activation of the NF-κB transcription factor, which induces the expression of cytokines and other OS-related proteins, which together cause cell death (100, 101). Due to polyunsaturated fatty acids incorporated into cell membranes, retinal cells are a good target for OS and have high oxygen consumption rates (101, 102). Some *in vitro* and *in vivo* studies showed the reduction of OS in the presence of RSV in diabetic retinopathy. It has been shown that RSV treatment of ARPE-19 cells decreases OS that was stimulated by a hypoxia-mimetic agent (88, 103). Soufi et al. designed a study to evaluate the possible effects of RSV application on OS and showed that 4 months oral RSV administration significantly reduces hyperglycemia and OS factors (GSSG/GSH ratio and lipid peroxidation index) in streptozotocin-nicotinamide-induced diabetic retinopathy rats (95). Li et al. used bovine retinal capillary endothelial cells to study the molecular mechanism of the RSV effect. They found that the hyperglycemia-induced enhancement of ROS was inhibited by RSV via the activation of the AMPK/Sirt1/PGC-1α pathway (104). It was found that RSV reduces intracellular ROS levels and endothelial-to-mesenchymal-transition in high glucose-exposed primary human retinal endothelial cells through suppression of PKC (105). Mohammad et al. found that increasing SIRT1 expression by RSV reduces the upregulation of HMGB1 in the diabetic retina. They suggested that the possible signaling cascade of this cross-talk plays a role in the regulation of BRB breakdown and OS (97). Abnormal production and enhanced release of VEGF are involved in the pathogenesis of diabetic retinopathy. VEGF is recognized as an angiogenic factor and is considered as a stimulator of angiogenesis in diabetic retinopathy (101). Hyperglycemia-induced angiogenesis leads to the production of leaky vessels, causing loss of retinal pericytes and vision. Available studies show that RSV treatment is effective as an antiangiogenic agent (106). Yar et al. investigated mRNA levels of VEGF, Angiotensin-converting enzyme (ACE), and Matrix Metalloproteinase-9 (MMP-9) in diabetic rat eye tissues after RSV treatment and did not find significant changes in their expression (107). One study by Kim et al. in mouse retinas of early diabetes showed the blocking effect of RSV on early vascular lesions and diabetes-induced VEGF levels (108). Michan et al. did not find significant repression of vascular pathologies after RSV treatment in a model of oxygen-induced retinopathy (109). Ruginǎ proposed polyelectrolyte microcapsules containing RSV as a delivery carrier with anti-VEGF effect in high glucose-exposed primary human retinal pigmented epithelial cells (110). The studies that were performed based on the beneficial effects of RSV on diabetic retinopathy are listed in Table 2.

**Table 2:** List of studies on diabetic retinopathy by resveratrol

Resveratrol (dosage/duration)	Study type and model	Findings	Ref.
**- 5 and 10 mg/kg/day** **- 30-, 32-, 34-, and 36-weeks**	**In-vivo** - Diabetic Sprague–Dawley rats	- RSV reduces retinal neural apoptosis in diabetic retina	(91)
**- 50 mg/kg/orally** **- 7 days**	**In-vivo** - C57BL/6 mice with STZ-induced diabetes	- Anti-inflammatory effect of RAV is represented via AMPK activation - RSV reverses retinal AMPK dephosphorylation, SIRT1 deactivation, and NF-κB phosphorylation	(90)
**Trans-RSV** **- 5 mg/kg/day** **- 30 days**	**In-vivo** - STZ-induced diabetic rats	- Trans-RSV-induced changes in gene expression level do not appear to affect functions of retinal pigment epithelium - Findings suggest normalization of apoptosis-related proteins and ERK1	(92)
**In-vitro** **- 10, 20, or 30 mmol/l** **- at least 3 days** **In-vivo** **- 5 and 10 mg/kg/day treatments** **- 1, 3, 5, and 7 months (72 h after STZ)**	**In-vitro** - Rat Müller cells **In-vivo** - STZ-induced diabetic rats	- This study showed the anti-apoptosis effects of resveratrol administration *in vitro* and *in vivo* - RSV treatment inhibited cell apoptosis via miR-29b. MiR-29b/specificity protein 1 (SP1) pathway is involved in the inhibition of apoptosis - The plasma levels of glucose and fructosamine in RSV-treated diabetic rats significantly decreased	(93)
**In-vitro** **- 10, 50, 100, 200, and 500 µM** **- 24 h** **In-vivo** ***A subgroup STZ-diabetic rats:*** **- 0.1 μg/mL or 1 μg/mL in one eye** **- 7 days (retinal vascular permeability analysis)** **- 24 h (mRNA expression analysis)** ***Other rats:*** **- 5, 10, or 50 µg/kg/day** **- 12 weeks**	**In-vitro** - Rat retinal endothelial cell (RREC) **In-vivo** - STZ-induced diabetic rats	**In-vitro Results** - RSV has been shown to play a protective role against high glucose-induced damage and inflammation via PON1 - RSV decreases the activation of caspase 3 and synthesis of Ox-LDL in high glucose-stimulated RRECs **In-vivo Results** - RSV prevented retinal apoptosis, retrieved the insulin level, induced PON1 expression and activity, and decreased the retinal vascular permeability, retinal damage, retinal AGEs, Ox-LDL, and inflammatory cytokines in STZ-diabetic rats	(94)
**- 5/mg/kg/day** **- 4 months**	**In-vivo** - STZ-induced diabetic rats	- RSV showed several beneficial properties, including antihyperglycemic, anti-apoptosis, antioxidant, anti-inflammatory effects, and reduction of the NF-kB activation	(95)
**- 5/mg/kg/day** **- 4 months**	**In-vivo** - STZ-induced diabetic rats	- RSV reduced the elevated levels of plasma inflammatory markers, including TNFα, IL-6, and NF-κB - It showed antihyperglycemic, anti-apoptosis, and antioxidant effects	(96)
**- 50 mg/kg (immediately after establishment of diabetes, second injection after 2 weeks, and retina isolation after 4 weeks)**	**In-vivo** - STZ-induced diabetic rats	- RSV prevented diabetes-induced reduction of SIRT1 and upregulation of HMGB1 and RAGE - Moreover, it significantly increased the occludin expression and decreased diabetes-induced breakdown of blood-retinal barrier	(97)
**- 5 mg/kg/day** **- 4 months**	**In-vivo** - STZ-induced diabetic rats	- RSV prevented the increase of apoptosis and pro-inflammatory mediators (IL-6, TNF-α, and COX-2). It attenuated the NF-κB activation and mRNA expression level	(98)
**- 1.25, 2.5, 5, and 10 µM** **- 9 days**	**In-vitro** - Retinal pigment epithelial cells (ARPE-19)	- Trans-RSV prevented secretion of VEGF, TGF-β1, COX-2, IL-6, and IL-8. It inhibited PKCβ activation, and Cx43 downregulation and increased GJIC.	(99)
**- 20 µM** **- 24 h or 48 h**	**In-vitro** - ARPE-19	- RSV significantly attenuated the extracellular release of HMGB1 from cells under oxidative and/or hypoxic stresses	(103)
**- 1, 5, 10, or 20 µM** **- 48 h**	**In-vitro** - Bovine retinal capillary endothelial cells (BRECs)	- RSV attenuates intracellular ROS via AMPK/Sirt1/PGC-1*α* pathway and represses apoptosis process	(104)
**- 1 µM** **- 24 h**	**In-vitro** - Primary Human Retinal Endothelial Cells (HRECs)	- RSV attenuated ROS production and epithelial-mesenchymal transition (EMT) in high glucose-exposed cells	(105)
**- 10 mg/kg/day** **- 4 continuous weeks (4 weeks after STZ injection)**	**In-vivo** - STZ-induced diabetic rats	- RSV repressed the endothelial nitric oxide synthase (eNOS) expression, whereas that expression of VEGF, ACE, and MMP-9 remained unchanged	(107)
**- 20 mg⁄kg, daily** **- 4 weeks (1 month after STZ injections)**	**In-vivo** - STZ-induced diabetic mice	- RSV blocked diabetes-induced vessel leakage, pericyte loss, and VEGF expression level in the mouse retinas	(108)
**- 400 mg/kg body weight, given daily from postnatal to 5 to 17 days**	**In-vivo** - OIR (oxygen-induced retinopathy)-diabetic mice	- RSV treatment did not show a protective effect against oxygen-induced retinopathy	(109)
**Polyelectrolyte microcapsules loaded with RSV** **- 24 h treatment**	**In-vitro** - Retina pigmented epithelial D407 cells	- A polyelectrolyte microcapsule loaded with RSV was designed that could be used as a potential intraocular RSV-delivery system for anti-VEGF therapy	(110)

### RSV and diabetic neuropathy

Diabetic neuropathy is a drastic long-term and the most frequent peripheral neuropathy associated with DM. Diabetic neuropathy is a usual cause of non-traumatic amputation, affecting 50–60% of patients with diabetes. Neuropathy is defined as the progressive destruction of the nerves. It is heterogeneous and affects different parts of the nervous system. Depending on the location and type of nerve fibers, various clinical manifestations occur. This is a late complication of type 1 diabetes and an early complication in T2DM. Diabetic neuropathy symptoms depend on the nervous injury location and can include motor alterations, such as weakness, sensory symptoms, tingling, pain, or autonomic changes such as urinary symptoms (111). Current therapies for diabetic neuropathy are challenging, mainly due to their side effects. Therefore, there is an increasing need of introducing novel, safe, and effective agents for diabetic neuropathy therapy. The pathophysiology of diabetic neuropathy involves a complex cascade of specific interconnected molecular pathways. It is reported that OS and pro-inflammatory cytokines are the major factors contributing to the pathophysiology of diabetic neuropathy (112, 113). Peripheral diabetic neuropathy has been directly associated with the increase in ROS and OS. This OS condition finally leads to vascular impairment, endoneurial hypoxia resulting in impaired neural function, and reduced conduction velocity (114, 115). Long-term hyperglycemia induces OS, leading to the activation of transcription factor NF-κB in peripheral neurons. Increased NF-κB mediates the production of pro-inflammatory cytokines (including iNOS, TNF-α, IL-6, and COX-2) that drive nerve damage (60, 67).

In the last decade, growing attention has been paid to herbal medicine and polyphenols, and study findings have indicated their association with decreased incidence of DM and its related complications (116, 117). RSV polyphenol has a potential neuro-protective effect in diabetic neuropathy and could be applied as an alternative therapy (118, 119). Many studies have investigated the effect of RSV on diabetic neuropathy and the mechanisms involved in its healing property. Recently, it has been suggested that RSV exerts neuro-protective effects via the activation of SIRT1, an enzyme that deacetylates regulatory proteins in the cells (120). Many studies have indicated the anti-inflammatory and antioxidant effects of RSV in improving diabetic neuropathy. Production of hyperglycemia-related ROS is directly linked to the diabetic neuropathy pathogenesis by triggering the production of IL-1*β* and TNF-*α*. In addition, IR and hyperglycemia in the central nervous system (CNS) are associated with the TNF-*α* signaling pathway, which result in pain and hyperalgesia in DN (121). RSV, via preventing the inflammatory response and proinflammatory cytokines, can be a promising agent in managing neuropathy-mediated damaging pathways. Another study reported that expression levels of xanthine oxidase (XO), nitric oxide (NO), and malondialdehyde (MDA) were elevated in STZ-induced animal models of diabetes. They showed that RSV therapy for 6 weeks significantly reduced the ROS and enhanced glutathione level (122). Additionally, DM by OS leads to enteric nervous system damage. On the other hand, higher levels of lipid hydroxide and NO are described in diabetic rats, where RSV diminishes the OS level to normal states; therefore, it was concluded that RSV plays an important role in the treatment of diabetic neuropathy through extinguishing the extreme ROS (123). Zhang et al. reported that the anti-inflammatory effect of RSV attenuates the severity of diabetic neuropathy by protecting peripheral nerves from apoptosis by inhibiting the NF-KB pathway and increasing Nrf2 expression (124). Another study was conducted in diabetic neuropathy rats models about the RSV effect on nerve function, OS, and DNA fragmentation (125). The authors demonstrated that RSV of 10 and 20 mg/kg produces significant improvement in the nerve conduction deficits by 82 and 90%, respectively, and in NBF deficits by 71 and 92%, respectively, in diabetic rats (125). In addition, they suggested that the reduction in OS and DNA fragmentation may be the potential protective effect of RSV in the treatment of diabetic neuropathy (125). Recently, it was described that AMPK, as a cellular energy homeostasis regulator, has a main role in DN (126). Investigations indicated that the suppression of AMPK activity was associated with OS and inflammatory response (127). In addition, RSV was known as a potential antioxidant and also hypoglycemic agent through the AMPK-stimulating effect (75, 128). A research by Chang et al. reported that RSV treatment remarkably reduced the expression of AMPK protein and its phosphorylation compared with non-treated diabetic group (49). These findings propose that RSV, with protective effects against OS and enhanced expression and activation of AMPK, exerts its beneficial effects on diabetic neuropathy (49). Saed et al. evaluated anti-neuropathy properties of RSV and its effect on SIRT-1 expression in animal models and found that receiving 200 mg/kg RSV per day significantly reduced blood sugar level and improved peripheral neuropathy, which was compared with metformin (*P* < 0.01). Moreover, SIRT-1 expression was increased in the treated group with RSV (129). Recently, Gonzalez et al. investigated the efficacy of daily RSV treatment in the neuromotor impairment and hearing loss in a mouse model of diabetic neuropathy (111). The results indicated that RSV treatment reduced plasma inflammatory biomarkers and Schwann cell demyelination, improved attenuated neuromotor and electrophysiological impairment, and reduced sciatic nerve histological anomalies and hearing loss in diabetic mice (111). According to Wang et al., the combination effect of mesenchymal stem cells (MSCs) and RSV on type 1 diabetic neuropathy impressively improved the levels of blood glucose, C-peptide, NGF, MBP, and NF-κB in mice. In addition, the greatest diameter of the axon and the number of myelinated nerve fibers were observed in the examined group (130). Accordingly, the combined therapy with RSV and MSCs could be a novel and potential therapy in diabetes-induced neuropathy. All the findings from literature clearly showed that RSV demonstrates neuro-protective action in patients with DN. Table 3 summarizes the available studies and mechanisms of action of RSV in diabetic neuropathy treatment. Diabetic neuropathic pain (DNP), as an intractable complication of DM, affects ~30% of diabetic patients (131), resulting in the dysfunction of peripheral nerves, including degeneration, allodynia, hyperalgesia, and insensitivity of nerves (132). In addition, thermal hyperalgesia and mechanical allodynia are known as the most common forms of DNP. In 2020, Cui et al. investigated the effects of RSV on diabetic mechanical allodynia (DMA)-related behaviors in rats (133). The results confirmed that the RSV had a dose-dependent analgesic effect on DMA model rats. In addition, downregulation of P2X3R expression in the dorsal root ganglion (DRG) and spinal dorsal horn (SDH) might be the potential mechanism of RSV analgesic effects on DMA-related behaviors (133).

**Table 3:** Available studies of resveratrol effects in diabetic neuropathy

Herb (dosage/duration)	Study type and model	Finding	Ref.
***In diabetic mice:*** - 100 µg/Ml in water - 8 weeks ***Nerve injury model***: - Subcutaneously injected at 10 mg/kg/daily - 4 days	**In-vivo** - Nerve injury model (male C57BL/6J mice) and male db/db diabetic mice	- Daily RSV treatment partially reduced the pathological phenotype and the neuromotor impairment in neuropathy animals induced by nerve injury - RSV improved neuromuscular performance and nerve conduction and restored myelin structure and hearing system - These findings suggest the efficacy of RSV in neuropathy disorder induced by diabetes	(111)
- 10 mg/kg/daily - 6 weeks	**In-vivo** - STZ-induced diabetic rats (60 mg/kg body weight)	- Treatment with RSV reduced MDA, XO, and NO production and increased glutathione levels - This study demonstrates that RSV is a potent neuroprotective agent against diabetic oxidative damage	(122)
- 10 mg/kg - 120 days	**In-vivo** Male Wistar rats STZ-induced diabetics rats	- RSV contributed in attenuation of neuronal loss - The results demonstrated that RSV has antioxidant and neuroprotective effects in myenteric plexus in rats with DM	(123)
- 10 ml/kg 10% - Once a day - 12 weeks	**In-vivo** - STZ-treated model mice (120 mg/kg body weight 1% STZ)	- The pain and temperature sensitivities of diabetic mice were improved - Nrf2 expression was increased in the diabetic peripheral nerves, and NF-KB pathway inhibition protected nerves upon RSV treatment in peripheral neuropathy - RSV modulated the anti-inflammatory microenvironment of peripheral nerves by increasing Nrf2 activation and p-p65 expression	(124)
- 10 and 20 mg/kg, i.p./daily - 2 weeks	**In-vivo** - STZ-diabetic (55 mg/kg I.P) rats developed neuropathy	- Significantly ameliorated the alterations in MNCV, NBF, and hyperalgesia - RSV attenuated enhanced levels of MDA, peroxynitrite and produced increase in catalase levels in diabetic rats - Neuroprotective effect of RSV mediated through reduction in OS and DNA fragmentation	(125)
- 25, 100, and 400 mg/kg	**In-vivo** - I.P injection of 60 mg/kg STZ	- RSV downregulated P2X3R expression in the DRG and SDH, which may be potential analgesic mechanism of RSV on DMA-related behaviors	(133)
- 20mg/kg/day - 6 weeks	**In-vivo** - STZ-induced mice (45 mg/kg body weight)	- Decreased cerebral MDA, COX-2, and concentrations of XO and NO - Increased cerebral level of IL-4 and GSH in the cerebral and cortex	(134)
**RSV+ 4-ANI** - RSV (10 mg/kg) - 4-ANI (3 mg/kg) - 2 weeks	**In-vivo** - Rats with type 1 diabetes induced by STZ at a dose of 55 mg/kg, rat	**- RSV+ 4-ANI** 1) Attenuated conduction and nerve blood flow deficits and ameliorated neuropathic pain 2) Reversal of biochemical alterations (peroxynitrite, MDA, and NAD levels) 3) PAR accumulation in the sciatic nerve	(135)
- Orally/ 10 and 20 mg/kg - 2 weeks	**In-vivo** - Type 1 diabetic rats by STZ at a dose of 55 mg/kg (i.p.)	- RSV decreased the expression of p65 and IκB-α in treated rats - Ameliorated the elevated levels of TNF-α, IL-6, and COX-2 - RSV decreased in nerve MDA levels - NF-κB inhibitory activity and anti-inflammatory activity of RSV contribute to neuroprotection in diabetic neuropathy	(136)
**RSV:**	**In-vivo** - Type 1 diabetic rats by single dose of 60 mg/kg STZ	- This study results suggest that modulation of the Sirt1/FoxO1 pathway may be a potentially useful therapeutic target for DN	(73)
- 10 mg/kg orally - 2 weeks	**In-vivo** - Diabetic rats by single I.P injection of STZ (65mg/kg)	- RSV significantly attenuated the cold allodynia and thermal hyperalgesia. - RSV introduced as an adjuvant therapy for the prevention and treatment of diabetic neuropathy	(137)
**RSV:** - 20 mg/kg/day **Curcumin:** - 60 mg/kg/day	**In-vivo** - STZ- induced diabetic mic (200 mg/kg)	- This study supported beneficial effect of these combinations with insulin in attenuating diabetic neuropathic pain via inhibition of NO and TNF-*α* levels	(138)
- 1–10 μM	**In-vitro** - Rat neuronal hippocampal cells	- The results indicate that PKCγ subunit plays a main role in neuronal integrity by RSV	(139)
- 5 mg/kg bodyweight/orally - 9 weeks	**In-vivo** - Male Sprague-Dawley rats & Swiss Webster mice - Type 1 diabetes by single I.P injection of 75–85 mg/kg or two injections of 90 mg/kg STZ	- RSV increases the level of AMPK in sensory neurons- RSV also contributed to reversal of thermal hypoalgesia and attenuation of foot skin intraepidermal nerve fiber loss	(140)
- DRG was treated with RSV in 1–25 μM - 1 h	**In-vivo** Sprague-Dawley rats	- 1 μM of RSV reduces the DRG neuron death from 70 to 50%, whereas 25 μM decreases the DRG neuron death to 42%	(141)
- 20 mg/kg intraperitoneally once daily - 4 weeks	**In-vivo** Male Wistar rats single dose of STZ- induced diabetic rats (55 mg/kg)	- RSV normalized diabetic malonedialdehyde and oxidized glutathione levels and strengthens the action of all antioxidant enzymes - The decrease in the requirement for the activation of antioxidant defense systems in the brain tissues of diabetic rats	(142)
- 80 mg/kg, 10 ml/kg - Once a day - 4 weeks	**In-vivo** STZ-induced diabetic rats (60 mg/kg)	- RSV ameliorates cognitive decline in STZ-induced diabetic model rats - The potential mechanism includes the inhibition of hippocampal apoptosis via the Bcl-2, Bax, and Caspase-3 pathways and improvement of synaptic dysfunction - BDNF plays an indispensable role in this mechanism	(143)
- 20 mg/kg/day intraperitoneally - 4 weeks	**In-vivo** - T2D rats induced by HFD-STZ (30 mg/kg) **HFD** (58% fat, 17% carbohydrates, 25% protein, 310 g/kg butter, 60 g/kg vitamins and minerals, 10 g/kg cholesterol, 1.0 g/kg yeast powder, 253 g/kg casein, and 1.0 g/kg sodium chloride)	- Memory impairment and disturbed insulin signaling in diabetic rats were reversed by RSV treatment partially via increased level of miRNA-21 - This study indicates role of miRNA-21 in modulating brain insulin signaling and hence alleviating cognitive dysfunction accompanying DM	(144)
- 200 mg/kg with I.P - Once a day - 14 consecutive days	**In-vivo** - Rat neuropathic pain model resulting from chronic constriction injury of the sciatic nerve	- Increased expression levels of IL-1RA and IL-1R2 - Increased IL-4Rα in dorsal spinal cord neurons - Results indicate that RSV enhances IL-4 receptor-mediated anti-inflammatory responses in the spinal cord and contribute to the alleviation of central sensitization in peripheral nerve injury	(145)
- 1 mg/kg - I.P. injected into the spinal cord of rats	**In-vivo** - Male Sprague–Dawley rats – OXA-induced neuropathic pain (4 mg/kg OXA)	- RSV reduced the levels of glial fibrillary acidic protein, TNF-α, IL-1β, and NF-Κβ. - RSV decreased COX-2 expression and suppressed ROS production - Totally, RSV reduced the spinal COX-2-mediated ROS generation and inflammatory response, suppressed astrocytic activation, and alleviated OXA-induced neuropathic pain.	(146)
- 2 mg/kg via oral gavage daily - 8 weeks	**In-vivo** Male Wistar rats via a single intravenous dose of STZ (65 mg/kg)	- RSV-mediated improvements in vascular and nerve function in old, obese, diabetic rats were associated with its reported antioxidant effects.	(147)

### RSV and diabetic foot ulcer

Diabetic foot ulcer is a disastrous DM complication and a remarkable cause of morbidity and mortality around the world (148). Diabetic foot ulcers are the most common non-traumatic risk factor for amputations in diabetic patients. According to the statistics, up to 20% of patients with diabetic foot syndrome need lower limb amputation during their disease (149), and 84% of all non-traumatic amputations are reported in these patients (150). Neuropathy, vascular occlusion, and secondary bacterial infection are the main events in the development of diabetic foot ulceration (DFU) (151). In fact, peripheral neuropathy with intrinsic muscle atrophy and functional anatomical changes in the foot can be involved in the development of DFU (152). Risk factors for DFU development are as follows: having diabetes for more than 10 years, male gender, and the presence of other complications of diabetes such as nephropathy, neuropathy, inflammation, the background of IR, and also history of foot ulceration (153). Diabetic neuropathy has several manifestations in feet, affecting sensory, motor, and autonomic fibers. In addition, inflammation and overproduction of pro-inflammatory cytokines including IL-6, IL-8, and TNF-α happen during foot ulceration development (154). Because of the increasing incidence of foot ulcers among diabetic population and lack of a pharmacological approach in controlling diabetic ulceration, there is a growing interest in the use of alternative herbal therapies for DFU such as RSV as a polyphenolic compound. Patients treated with RSV have shown reduced pro-inflammatory markers, such as IL-1β, IL-6, TNF-α, MMP-2, -3, -9, and C-reactive protein (CRP) (155, 156). The main reason for the persistence of inflammation in diabetics is the lack of timely polarization of pro-inflammatory macrophage (M1) to anti-inflammatory macrophage (M2). In normal physiological conditions, polarized macrophages, by secretion of VEGF, fibroblast growth factor (FGF), epidermal growth factor (EGF), and also cytokines, including IL-10 and IL-4, involve in the regulation of cell proliferation and differentiation near the ulcer (157). In diabetic patients, polarization of M1 into M2 does not happen quickly, which causes persistent inflammation condition and delayed wound healing (158). Therefore, stimulating and accelerating the macrophage polarization process can be a helpful therapy for diabetics. Numerous in-vitro and in-vivo studies confirmed the effects of RSV on switching from M1 to M2 phenotype macrophages in tissues (159). In addition, Ding et al. reported that RSV accelerates wound healing by inducing M2 macrophage polarization in diabetic mice (160). The authors showed that RSV significantly increased diabetic wound healing by a reduction in the secretion of inflammatory factors, including TNF-a, iNOS, and IL-1b, and promoted M2 macrophage polarization (160). However, the detailed mechanisms associated with RSV action in wound healing and prevention and management of diabetic ulcers are not completely understood. One of the most relevant pathways in this regard is related to SIRT1 signaling. Many SIRT1 activating compounds such as RSV were found to be protective, improving diabetic wound healing through the regulation of inflammation, cell migration, OS response, and the formation of granulation tissue at the wound site (161).

According to the literature, RSV acts as SIRT1-upregulator, which inhibits the inflammation cascade and elevates the cell survival around the wound site (162). By activating SIRT1, RSV inhibits pro-inflammatory IL-6, TNF-α, and IL-1β levels in serum, induces collagen synthesis, and also stimulates autophagy. Other RSV mechanism in wound healing is the regulation of VEGF expression, which has a broad spectrum of wound healing-related activities, ranging from capillary growth to increased cell migration, collagen deposition, and epithelialization. Additionally, this natural drug reduces OS by reducing AP-1 and COX2 and also increasing FOXO and Nrf2 factors (163). Recently, wound dressings have been developed that use natural and herbal materials in their construction. For instance, a study used RSV microparticles (Dermalix (Dx)) for diabetic foot ulcers, and the efficacy and safety of which was comparable to standard wound care (SWC) in diabetic patients (164). After a 4-week follow-up, they evaluated the wound area measurement, total collagen, VEGF, tumor necrosis factor (TNF), IL-1, Caspase-3, glutathione, reduced/oxidized glutathione, and lipid peroxidation levels. The percentage closures of wounds were significantly higher in the Dx group than the SWC group. Interestingly, Dx considerably affected the levels of TNF, caspase 3, and reduced/oxidized glutathione. In addition, Dx provided two fold faster wound healing and reduced OS. Finally, the authors concluded that the Dx usage in the first phase of wound would support the wound area healing with a faster and safe profile (164). In another study, Gokce et al. evaluated the effects of collagen-laminin dermal matrix impregnated with RSV loaded hyaluronic acid-DPPC microparticles in wound healing of diabetic rats and reported that this combination is a safe and promising choice for the treatment of diabetic wounds (165). However, therapeutic use of RSV in clinic for diabetic foot complications has not been reported, and therefore, further studies are required. The protective effects of RSV in DFU in animal, human, and in-vitro models are provided in Table 4.

**Table 4:** List of studies that used resveratrol for diabetic ulcer healing

Herb (dosage/duration)	Study type and model	Finding	Ref.
**- 50 mg/kg/day** **- 4 weeks**	**In-vitro** - HUVECs **In-vivo** - C57BL/6 mice (db/db & db/m)	**-** RSV accelerates the diabetic wound healing via its SIRT1-dependent pro-angiogenic effect **-** RSV activated endothelial SIRT1 and promoted FOXO1 degradation, which further de-repressed c-Myc expression against high glucose	(166)
**RSV-loaded hyaluronic acid-DPPC microparticles:** **- Range: 0.1–20 µg/ml** **- 14 days**	**In-vivo** - STZ-induced diabetic wound rats	- The wound was completely healed at the end of the 14th day following its induction in DM-MP-RSV group - Highest amount of collagen fibers and efficient re-epithelization in DM-MP-RSV group - Decreased SOD compared to diabetic wound control group and decreased GPx compared to diabetic control group	(165)
**- 10 µmol/L** **- 15 days**	**In-vivo** - STZ-induced C57/B6 mice **In-vitro** - Human monocyte line THP-1	**In-vivo** - Increased diabetic wound healing (*P* < 0.05) - Promoted the expression of α-SMA and collagen I (*P* < 0.05) - Reduced the secretion of inflammatory factors (TNF-α, iNOS, and IL-1β) (*P* < 0.05) - Promoted M2 macrophage polarization by increasing Arg-1 and CD206 expression (*P* < 0.01) **In-vitro** **-** Promoted the polarization of M2 macrophages (*P* < 0.001) and reduced the secretion of pro-inflammatory factors (TNF-α, IL-6, and IL-1β) (*P* < 0.05)	(160)
***Trans-*RSV:** **- 50 mg of trans-RSV or placebo capsules** **- 60 days (after a meal/morning and evening)**	**Clinical trial** **(**human study)	- Promoted reduction in ulcer size - Improved performance in the foot pressure test - Significant decline in the plasma fibrinogen level	(167)

### RSV in diabetic cardiomyopathy

Cardiovascular disorders are as one of the main complications of uncontrolled diabetes (168). The incidence of DM is increasing and has quickly become one of the most widespread chronic diseases worldwide. There is a close relationship between DM and cardiovascular disease (CVD), which is a most common cause of morbidity and mortality among patients with diabetes. The risk factors of CVD include obesity, hypertension, and dyslipidemia that are prevalent in diabetic patients and expose them at increased risk for cardiac disorders (169). CVD includes various disorders of the heart and blood vessels, such as peripheral artery disease, deep vein thrombosis, coronary artery disease (CAD), cerebrovascular disease, and congenital heart disease (170). Cardiomyopathy triggers structural and functional changes in cardiomyocytes that results in the induction of myocardial fibrosis. It is defined by the deposition of significantly high amounts of collagen and other extracellular proteins in the heart tissue (171). Certain metabolic disorders associated with adipose tissue, such as adipocyte dysfunction and its inflammatory effect, act as causal factors of T2DM, coronary atherosclerosis, dyslipidemia, and arterial hypertension, leading to cardiomyopathy (172). In diabetic patients, 55–85% of mortality is observed to be due to diabetic cardiomyopathy (DCM) (173). DCM has been described as a diabetes-mediated ventricular dysfunction, independent of any vascular disease and hypertension, manifesting initially by diastolic dysfunction, in the middle by systolic dysfunction, and finally by heart failure. DCM is a prevalent malady in the diabetic community as it is detectable even in approximately 60% of well-controlled type 2 diabetic patients (174). While various mechanisms have been discussed for the onset and advance of DCM, it is believed that OS resulting from diabetes-mediated hyperglycemia, mitochondrial uncoupling, and lipotoxicity plays central roles in the pathogenesis of DCM (175, 176). OS, an imbalance between oxygen/nitrogen-free radical production, and antioxidant defense cause oxidation or glycation of lipids, proteins, and DNA and result in ECM alteration, autonomic neuropathy, small vessel disease, and defects in cellular Ca^2+^ transport and contractile proteins. These complications, in turn, can result in stiffness, cardiac hypertrophy, fibrosis, and apoptosis (174, 177). As with most chronic inflammatory diseases, hyperlipidemia, inflammation, and hyperglycemia are the key initiating factors for DCM, which stimulate resident cardiomyocyte hypertrophy, fibroblast proliferation, dysfunction, and cardiac remodeling (178). Impaired insulin metabolic signaling, hyperglycemia-induced abnormal AGE/RAGE signaling, mitochondrial dysfunction, increased fatty acid utilization, and impaired calcium handling ER stress along with coronary endothelial dysfunction are considered pathogenic causes in T2DM-induced DCM (179–181). During recent years, many investigations have been directed toward increasing antioxidant defense as a novel therapeutic approach against diabetes-related cardiovascular complications (174, 175), and attention is also increased toward herbal medicines such as RSV. A great number of in-vivo studies have reported that RSV presents glucose-lowering effects in types 1 and 2 DM (182). RSV has been shown to provide multiple useful effects in CVDs, such as myocardial ischemia/reperfusion (I/R) injury, heart failure (183), and atherosclerosis (184). The wide-range cardioprotective effects of RSV have attracted the consideration of researchers in terms of its role in DCM prevention and treatment (185). The exact molecular mechanism of the effect of RSV on heart complications caused by diabetes has not been clarified, but there are possible mechanisms in this regard, which are mainly related to the antioxidant, anti-inflammatory, and antiapoptotic effects of RSV. Hyperglycemia-induced apoptosis participates in the pathogenic alterations in DCM. Sustained high glucose is a main cause of excess ROS production, dysregulation of lipid metabolism, IR, and disturbance of cytoplasmic calcium, and as a result, cell death, left ventricular (LV) remodeling, and finally heart failure occur (186). The PI3K pathway is the main pathway related to apoptosis. PI3K, with the activation of serine-threonine kinase (Akt), induces the downstream substrate phosphorylation (187) in GLUT4, mammalian target of rapamycin (mTOR), and the FoxO3 transcription factor. It is found that RSV therapy leads to inhibition of high glucose-related apoptosis associated with the PI3K/Akt signal pathway in neonatal rat ventricular myocytes and activates the insulin receptor and FoxO3a signaling pathway (46, 188). Wu et al. conducted a study to explore the effect and mechanism of action of RSV on cardiac function in DCM (189). The aforementioned study data suggested that RSV ameliorates cardiac dysfunction by inhibiting apoptosis via the PI3K/Akt/FoxO3a pathway in animal models of DCM. Therefore, RSV could be a novel therapeutic potential against DCM by inhibiting apoptosis via the PI3K/Akt/FoxO3a pathway (189). Additionally, studies reported that RSV induces the expression and activity of SIRT1 (one of its targets) and confers protection against ischemia/reperfusion injury in cardiomyocytes via the regulation of uncoupling protein 2 expression (190). Several studies have been conducted that show RSV effects on heart failure, including in-vivo, in-vitro, and human studies. A study by Dekkers et al. showed the cardioprotective effects of RSV on the heart of diabetic rats through the reduction of infarct size and apoptotic cell death (191). Another study showed that treatment with RSV (2.5 mg/kg/day orally for 2 weeks) induced GLUT4 translocation and glucose uptake in diabetic rat myocardium (192). On the other hand, Delucchi et al. reported that treatment with RSV (2.5 mg/kg/day for 8 weeks) improves diabetic heart function by reducing ventricular inflammation and remodeling (193). In human studies, Timmers et al. showed that treatment with RSV (50 mg/kg/day for 16 weeks) improves muscle mitochondrial respiration by AMPK activation, increases SIRT1 and PGC-1α protein levels and citrate synthase activity, and, finally, improves the performance of the heart in CVD (194). It was also shown that 1 month treatment with RSV (10 mg/kg/day) improves diabetic heart function by improved LV diastolic function, endothelial function, and decreased LDL-cholesterol level and leads to protection against unfavorable hemorheological changes measured in patients with CAD (195). In an in-vitro study conducted by Pankaj K et al. on H9C2 cell, it was reported that SIRT1 activation by RSV is associated with decreased NFkB-p65 activity and NOX transcription (196). Similarly, knockdown or inhibition of SIRT1 in H9C2 cells increased acetylation of NFkB-p65, K310, and H3K9 (197). Another study has proven that RSV in H9c2 cells activates SIRT1, thereby mediating PGC-1α deacetylation, improving and increasing mitochondrial function, and alleviating DCM injury (198). Wu et al. showed that RSV exhibited the antiproliferative effect on cardiac fibroblasts, mcsf, via inhibiting ROS/extracellular regulated kinase (ERK) pathway and ameliorated myofibroblast differentiation via suppressing ROS/ERK/TGF-β (199). The beneficial effects of RSV on both experimental and human models have been summarized in Table 5. However, these data support the beneficial effects of RSV and provide support for the future development of new therapeutics in patients with DCM in the clinic.

**Table 5:** List of studies of resveratrol that used for diabetic cardiomyopathy

Herb (dosage/duration)	Study type and model	Finding	Ref.
**- 2.5 mg/kg/day** **- 7 days**	**In-vivo** -STZ-induced (65 mg/kg) diabetic rats	- Reduce infarct size and apoptotic cell death - Increased the expression of Hsc70, HSPp6, GRP75, Prdx-1, and Prdx-3	(191)
**- 2.5 mg/kg/day; IP** **- 1, 3, and 8 weeks**	**In-vivo** STZ-induced (60 mg/kg) diabetic rats	- Prolongation of isovolumic contraction/relaxation times - Preserving the functional abilities of CSPCs and mature cardiac cells - Reduction inflammatory state and decreased unfavorable ventricular remodeling of the diabetic heart	(193)
**- 20 mg/kg/day by gastric gavages** **- 4 weeks**	**In-vivo** Type 2 diabetic (*Lepr^db^*) mice (strain: C57BLKS/J)	- Improves left ventricular (LV) diastolic relaxation - Increased expression of NO synthase (eNOS) - Reduction of O2·− production by inhibiting NADPH oxidase, gp91*phox* mRNA - Reduction of N-Tyr and iNOS expressions - Reduction of TNF-α mRNA	(200)
**- 5 mg/kg/day orally** **- 4 month**	**In-vivo** STZ-induced (50 mg/ /kg; IP) diabetic rats	- Improved LV diastolic pressure and circulatory proinflammatory markers - Improved left ventricular developed pressure - Reduction insulin concentration - Enhanced SOD activity and reduced GSSG/GSH ratio - Reduction of NF-kB	(201)
**- 5 mg/kg/day orally** **- 14 consecutive days**	**In-vivo** Zucker obese rats	- Attenuation of myocardial ischemic damage occurs by downregulating the expression of ET-1 - Increased expression of Glut4 - Reduction of endothelin expression and cardiac apoptosis - Recovery of CF, AF, and LVDP	(202)
**- Two different doses (1 or 5 mg/kg/day), IP injection** **- Randomized to 1, 3, or 6 weeks**	**In-vivo** STZ-induced (60 mg/kg) diabetic rats	- Improved the contractile efficiency of the diabetic heart - Reduction in blood glucose - Induce an almost complete recovery in baseline hemodynamic - Improved LV function	(203)
**- 40 or 100 μmol/L in transfected C2C12 cells** **- 4 g/kg in rat**	**In-vivo** - Rat - Human heart specimens (autopsy) **In-vitro** C2C12 cells	- Reducing expression levels of pro-hypertrophic markers ANP, BNP, β-MHC, MDA, and UCP2 - Increase of SOD, ATP content, mitochondrial DNA copy number, and expression of NRF - Increased expression of SIRT1 and increased PGC-1α deacetylation	(204)
**- 10 mg/day** **- 90 days**	**Clinical trials** Postinfarction patients with stable CAD	- Reduced LDL - Improved FMD and LV diastolic function - A trend toward improved LV systolic function	(195)
**- 30 μmol/L** **- 1 h**	**In-vitro** H9c2 cell	- Activation of SIRT1 - Reduction of NF-κB-p65 binding activity to DNA - Attenuates cardiac hypertrophy and OS via reducing NADPH transcription	(196)
**- 50 μmol/L** **- 48 h**	**In-vivo** DCM mouse model **In-vitro** High glucose (HG) cultured H9c2 cell	- RSV-induced SIRT1 activation ameliorates cardiac injuries in DCM through PGC-1α-mediated mitochondrial regulation	(197)
**- 50 mg/kg/day** **- 16 weeks**	**In-vivo** **-** High fat diet and STZ-induced diabetic rats	- RSV activates SIRT1, thereby mediating PGC-1α deacetylation, improving mitochondrial function, and alleviating DCM injury - SIRT1 induces SOD activation, which contributes to the alleviation of oxidative stress in a DCM heart	(198)
**- 2.5 mg/kg** **- 2 weeks orally**	**In-vivo** STZ-induced (65 mg/kg, IP) diabetic myocardium rats	- RSV activates AMPK/eNOS/NO, AMPK/Akt, Cav-1/eNOS pathways, and Cav3 expression, thus augmenting Glut-4 translocation to cell surface and glucose uptake during hyperglycemia	(205)
**- 10 μmol/L** **- 2 h**	**In-vivo** STZ-induced (50 mg/kg) diabetic rats **In-vitro** Rat derived H9C2 cardiomyocytes	- RSV activates SIRT1 and attenuates ER stress-induced cardiomyocyte apoptosis via PERK/eIF2α, ATF6/CHOP, and IRE1α/JNK-mediated pathways	(206)
**- Under 100 mg/kg/day**	**In-vivo** STZ-induced (150 mg/kg, IP) type 1 diabetic rats	- RSV enhances SERCA2a expression and augments cardiomyocyte Ca2+ homeostasis by activating SIRT1, thereby inducing cardioprotection in DCM heart	(207)
***Group 1:* about 60 mg/kg/day** ***Group 2:* about 300 mg/kg/day**	**In-vivo** STZ-induced (50 mg/kg/5 consecutive days) diabetic rats	- RSV promotes cardiomyocyte autophagic flux via SIRT1/FOXO1/Rab7 axis, which subsequently attenuates cardiomyocyte apoptosis and oxidative stress injury in diabetic state	(208)
**- 80 mg/kg** **- 12 weeks**	**In-vivo** STZ-induced (45 mg/kg) diabetic rats	- Inhibition of inflammatory factors, such as TNF-α, IL-6, and IL-1β - Downregulation of AT1R-ERK/p38 MAPK signaling pathway	(209)

## Conclusions

Due to the ever-increasing incidence of diabetes worldwide, it is very important to find a suitable treatment for the complications caused by DM. Common antidiabetic drugs are classified into glucosidase inhibitors, sulfonylureas, biguanides, and glinides, which mainly regulate blood glucose individually or in combination. Recently, natural compounds for the prevention and treatment of various diseases, especially chronic diseases, have attracted more attention. RSV as a natural polyphenol is present in different human dietary sources. A broad range of investigations on the beneficial roles of RSV is performed in-vitro and in animal model experiments. According to the literature, RSV exerts its effects via antioxidant and anti-inflammatory properties, the protection of islet β cells, the regulation of lipid metabolism, etc. RSV can improve diabetes and its complications through the regulation of several signaling pathways, mainly the SIRT1 and AMPK pathways. RSV effects have been widely reported in animal experiments; however, more studies in the field of administration strategies, for example, dosage of RSV, duration of treatment, and also the use of type of RSV derivative, must be performed to validate this trend. Overall, more clinical and observational data are necessary for understanding the mechanisms of RSV’s therapeutic potential. Taken together, further articles should study whether RSV is more efficacious in types of diabetic complications with different geographical areas and the duration of the disease. In addition, more studies should focus on emerging an RSV derivative with more bioavailability. Therefore, further studies are needed to establish the use of RSV for treating diabetes complications in humans.

## Data Availability

The data used to support the findings of this study are included within the article. Additional information can be requested by contacting the corresponding author.
